# Why Are Nigeria-Cameroon Chimpanzees (*Pan troglodytes ellioti*) Free of SIVcpz Infection?

**DOI:** 10.1371/journal.pone.0160788

**Published:** 2016-08-09

**Authors:** Sabrina Locatelli, Ryan J. Harrigan, Paul R. Sesink Clee, Matthew W Mitchell, Kurt A. McKean, Thomas B. Smith, Mary Katherine Gonder

**Affiliations:** 1 Unité Mixte Internationale 233, Institut de Recherche pour le Développement, INSERM U1175, and University of Montpellier, 34394 Montpellier, France; 2 Department of Biological Sciences, University at Albany – State University of New York, Albany, NY, 12222, United States of America; 3 Center for Tropical Research, Institute of the Environment and Sustainability, University of California, Los Angeles, 621 Charles E. Young Drive South, Los Angeles, CA, 90095, United States of America; 4 Department of Biology, Drexel University, Philadelphia, PA, 19104, United States of America; Centers for Disease Control and Prevention, UNITED STATES

## Abstract

Simian immunodeficiency virus (SIV) naturally infects two subspecies of chimpanzee: *Pan troglodytes troglodytes* from Central Africa (SIVcpz*Ptt*) and *P*. *t*. *schweinfurtii* from East Africa (SIVcpz*Pts*), but is absent in *P*. *t*. *verus* from West Africa and appears to be absent in *P*. *t*. *ellioti* inhabiting Nigeria and western Cameroon. One explanation for this pattern is that *P*. *t*. *troglodytes* and *P*. *t schweinfurthii* may have acquired SIVcpz after their divergence from *P*. *t*. *verus* and *P*. *t*. *ellioti*. However, all of the subspecies, except *P*. *t*. *verus*, still occasionally exchange migrants making the absence of SIVcpz in *P*. *t*. *ellioti* puzzling. Sampling of *P*. *t*. *ellioti* has been minimal to date, particularly along the banks of the Sanaga River, where its range abuts that of *P*. *t*. *troglodytes*. This study had three objectives. First, we extended the sampling of SIVcpz across the range of chimpanzees north of the Sanaga River to address whether under-sampling might account for the absence of evidence for SIVcpz infection in *P*. *t*. *ellioti*. Second, we investigated how environmental variation is associated with the spread and prevalence of SIVcpz in the two chimpanzee subspecies inhabiting Cameroon since environmental variation has been shown to contribute to their divergence from one another. Finally, we compared the prevalence and distribution of SIVcpz with that of Simian Foamy Virus (SFV) to examine the role of ecology and behavior in shaping the distribution of diseases in wild host populations. The dataset includes previously published results on SIVcpz infection and SFVcpz as well as newly collected data, and represents over 1000 chimpanzee fecal samples from 41 locations across Cameroon. Results revealed that none of the 181 *P*. *t*. *ellioti* fecal samples collected across the range of *P*. *t*. *ellioti* tested positive for SIVcpz. In addition, species distribution models suggest that environmental variation contributes to differences in the distribution and prevalence of SIVcpz and SFVcpz. The ecological niches of these two viruses are largely non-overlapping, although stronger statistical support for this conclusion will require more sampling. Overall this study demonstrates that SIVcpz infection is absent or very rare in *P*. *t*. *ellioti*, despite multiple opportunities for transmission. The reasons for its absence remain unclear, but might be explained by one or more factors, including environmental variation, viral competition, and/or local adaptation—all of which should be explored in greater detail through continued surveillance of this region.

## Introduction

**Fig 1:**
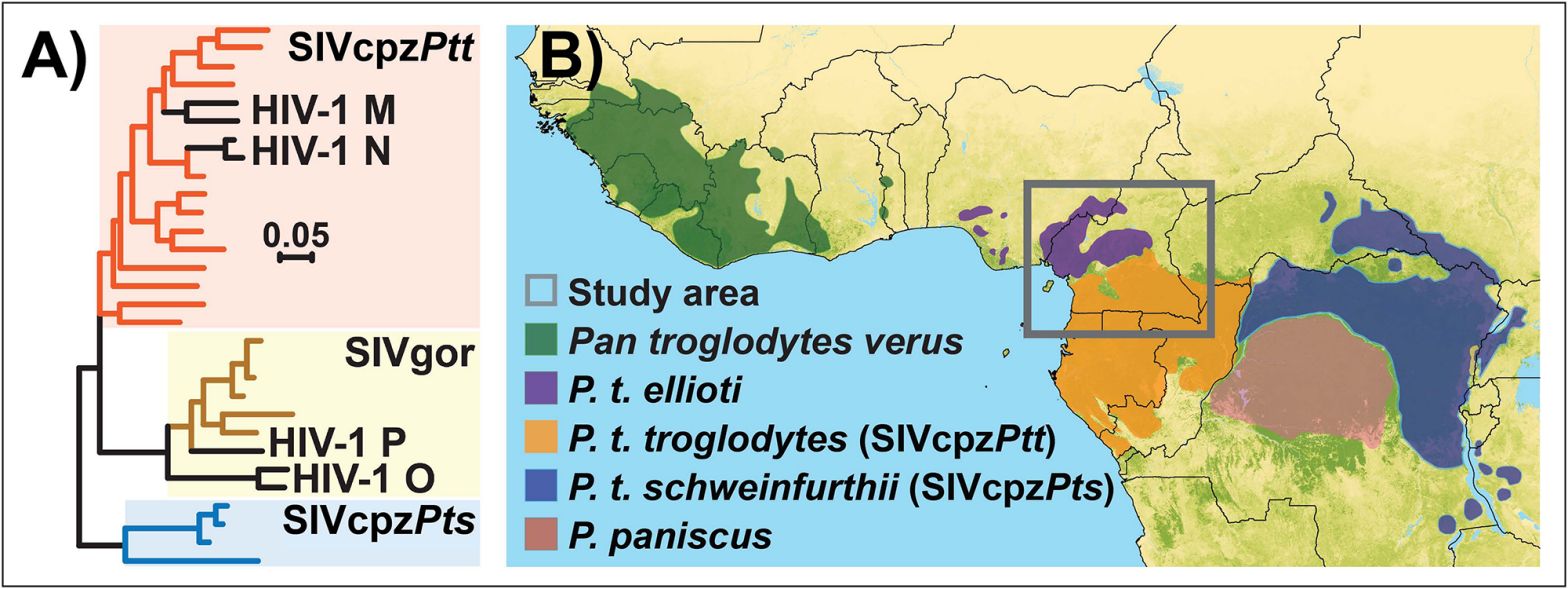
(A) Phylogeny of SIVs and HIVs infecting ape species. (B) Distribution of the genus *Pan* and SIVcpz viruses. The map background layer is “percent tree cover” from the MODIS satellite [81].

In chimpanzees, Simian Immunodeficiency Virus (SIVcpz) naturally infects only *Pan troglodytes troglodytes* from Central Africa (SIVcpz*Ptt*) and *P*. *t*. *schweinfurthii* from East Africa (SIVcpz*Pts*). SIVcpz*Ptt* was passed from chimpanzees to humans in southern Cameroon, giving rise to the HIV-1 group M pandemic and to HIV-1 group N [1,2]. HIV-1 group O and P also arose from transmission from *P*. *t*. *troglodytes* to gorillas before subsequent transmission to humans [3] (Fig 1A). SIVcpz infections are conspicuously absent in the two other chimpanzee subspecies: *P*. *t*. *verus* from West Africa and *P*. *t*. *ellioti* inhabiting Nigeria and western Cameroon, north of the Sanaga River [4,5,6] (Fig 1B).

Previous research proposed that differences in the occurrence of SIVcpz viruses among chimpanzee subspecies are the result of the evolutionary diversification of the subspecies and their subsequent demographic histories [7]. Specifically, *P*. *t*. *troglodytes* may have acquired SIVcpz after splitting from *P*. *t*. *verus* and *P*. *t*. *ellioti* through cross-species transmission and recombination of two lineages of SIVs infecting monkey species upon which chimpanzees prey [8]. Subsequently, SIVcpz likely spread eastward, although it remains unclear whether this occurred during or after the divergence of *P*. *t*. *troglodytes* from *P*. *t*. *schweinfurthii* [9,10]. Available genetic data provide some support for this latter hypothesis: *P*. *t*. *schweinfurthii* split recently from *P*. *t*. *troglodytes* [11,12] and may exchange an estimated 1.5 migrants per generation [13,14,15] (with a generation time range estimated to be between 22.5 and 28.9 years in chimpanzees [16]). However, this scenario also requires a preceding separation of *P*. *t*. *troglodytes* from *P*. *t*. *ellioti* and from *P*. *t*. *verus*, and no migration between them after their separation. Numerous genetic analyses suggest that *P*. *t*. *verus* separated from the other subspecies 0.5–1 million years ago with little to no migration with the other subspecies after its separation [11,12,13,17]. Furthermore, both whole genome analysis of multiple individuals from each subspecies [15], as well as the analysis of mitochondrial genomes [18], suggest the various subspecies split early in the species history into a western African group (*P*. *t*. *verus* and *P*. *t*. *ellioti*) and central/eastern African group (*P*. *t*. *troglodytes* and *P*. *t*. *schweinfurthii*). *P*. *t*. *verus* split from *P*. *t*. *ellioti* shortly thereafter with little evidence of migration between them afterwards, thus leaving no route for the horizontal spread of SIVcpz to *P*. *t*. *verus*.

It remains unclear whether long-term population isolation can also explain the absence of SIVcpz in *P*. *t*. *ellioti*, since opportunities exist for gene flow and virus exchange where its range abuts that of SIVcpz*Ptt*-positive chimpanzees in southern Cameroon. Recent population genetic studies showed that *P*. *t*. *ellioti* and *P*. *t*. *troglodytes* last shared a common ancestor approximately 200–500 kya [13,15,19,20]. Since this separation, these subspecies have exchanged between one and two migrants per generation, indicating that other factors leading to local adaptation have played a dominant role in their divergence [19]. Environmental variation has been shown to be important in not only driving the divergence of *P*. *t*. *ellioti* from *P*. *t*. *troglodytes*, but also between *P*. *t*. *ellioti* populations occupying the dense forests of western Cameroon from a population that occupies the mosaic woodland-savanna habitats of central Cameroon [21,22]. Different habitat types (rainforests versus drier, mosaic habitats) also influence group size, home range, feeding ecology and mating behavior [23,24,25,26]. Rainforests tend to have clumped, more easily defendable resources, whereas mosaics habitats tend to have more dispersed resources and increased seasonal fluctuations in fruit availability [27]. This can have an impact on the degree of food competition and antagonistic behavior, as well as on mating strategies in chimpanzees. SIV is transmitted primarily through sexual contact, whereas other viruses can spread via saliva or feces of infected individuals. The Simian Foamy Virus (SFV), for example, is a virus that can be spread during aggressive behavior through biting [28]. It is a species-specific virus known to infect populations of all four subspecies of chimpanzees at rates varying from 44 to 100% in the wild [29] and seems to be non-pathogenic in chimpanzees and other primates [30,31]. While prevalence of SFV for wild *P*. *t*. *ellioti* has been reported to be between 81% and 100% (between 44% and 100% for wild *P*. *t*. *troglodytes*) [29], only a few studies have attempted to quantify the prevalence of SIVcpz in *P*. *t*. *ellioti* from a small cluster of locations and mostly from a region just north of the Sanaga River [5,32].

The goals of this study were three fold: 1) to collect evidence, if any, of SIVcpz infections in a larger, more representative sample from across the range of *P*. *t*. *ellioti* in Cameroon; 2) to investigate the possibility that environmental variation, including climatic factors, topographic factors, and vegetation may have played a role in determining the distribution of SIV in the two chimpanzee subspecies inhabiting Cameroon, and 3) to compare and contrast viruses with different prevalence patterns and routes of transmission to understand how ecological variables may shape their distributions in wild host populations. To address this last aim, we examined the prevalence and distribution of an additional virus, SFV, in wild chimpanzees (SFVcpz).

## Materials and Methods

### Study sites, samples collection and shipment

**Fig 2:**
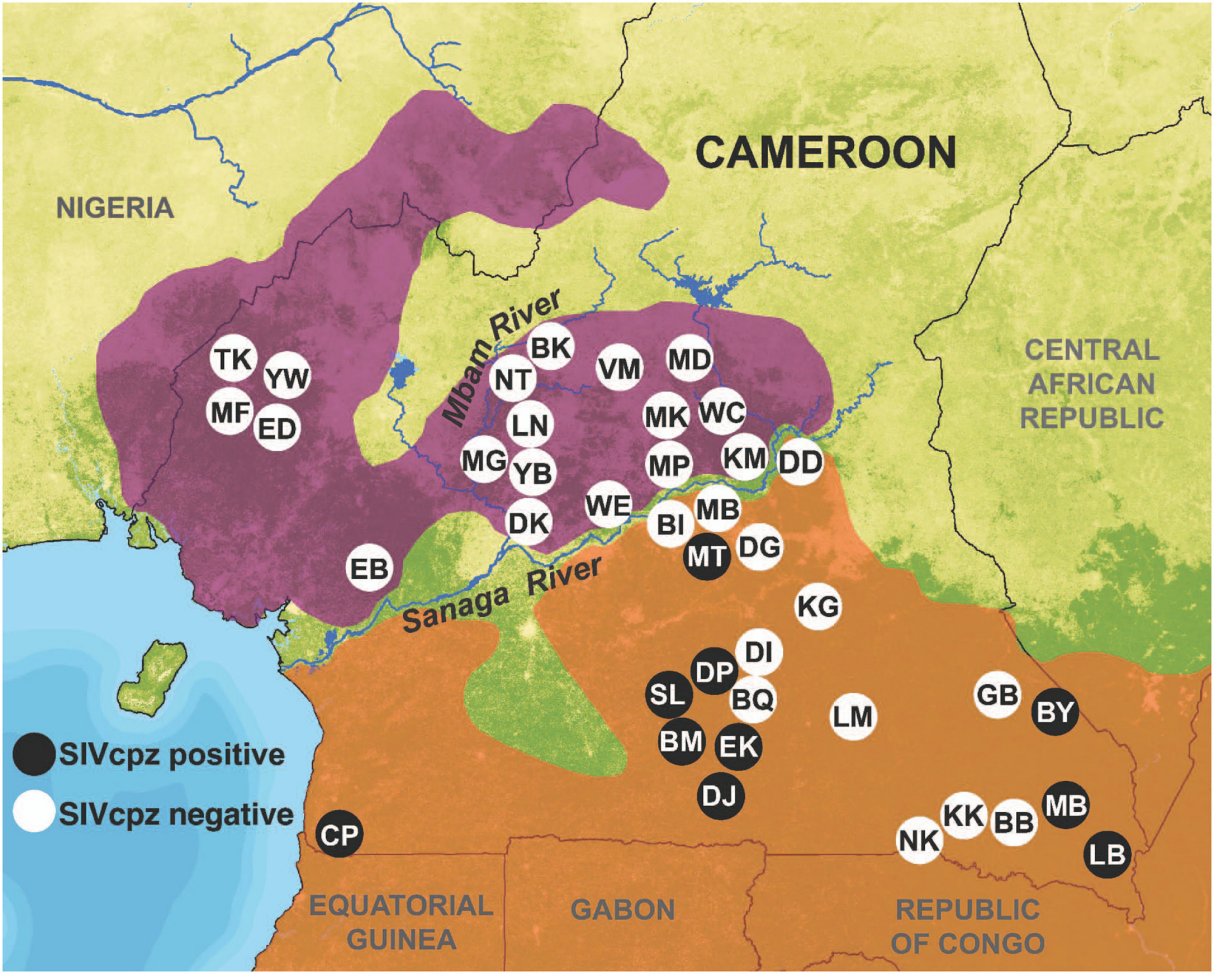
Chimpanzees sample locations. The locations are plotted against the distributions of *P*. *t*. *ellioti* (purple) and *P*. *t*. *troglodytes* (orange). SIVcpz positive locations are shown in black and SIVcpz negative locations are shown in white. The map background layer is “percent tree cover” from the MODIS satellite [81]. Rivers are from the HydroSHEDS hydrography layer [82].

We obtained permits from the Ministère des Forêts et de la Faune (MINFOF) and the Ministère de la Recherche Scientifique et de l’Innovation (MINRESI) in Cameroon. All samples were transported from Cameroon to the United States in accordance with Convention of International Trade in Endangered Species of Wild Fauna and Flora (CITES) and Center for Disease Control (CDC) export and import regulations. This research was carried out with IACUC approval from the University at Albany–State University of New York. In total, 41 locations were sampled across Cameroon: 18 sites inhabited by *P*. *t*. *ellioti*, and 23 sites inhabited by *P*. *t*. *troglodytes* (Fig 2). Included in these samples were those collected from 18 sites between 2009 and 2010 and chimpanzee fecal samples collected and analyzed between 2004 and 2007 by the research teams of Beatrice Hahn and Martine Peeters [2,5] (GPS coordinates of the sites sampled are listed in S1 Table). We recruited a team of ~9 field assistants for each mission and collected fecal material in the vicinity of forests were chimpanzee presence was reported by local hunters and villagers. We initially walked transects, hunters’ paths, or elephant paths in search of chimpanzee presence (e.g. night nests, footprints, and vocalizations). The Campo Ma’an (CP), Deng Deng (DD), Diang (DG), Mbam et Djerem (MD) and Mone (YW) sites are located in National Parks or forests reserves, whereas the remaining field sites are located in unprotected forests (Fig 2). Fecal samples were identified and collected by experienced trackers or researchers who estimated their likely time of deposition and placed 15–20 g of feces into 30 or 50 mL tubes and mixed with an equal volume of RNA*later*^*®*^ (Ambion, Austin, TX). Fecal samples were kept at ambient temperature for no longer than 2 weeks and subsequently stored at -20°C once returned from the field. Samples were shipped to the United States at ambient temperature, then stored at -20°C upon receipt. All the samples we collected were genotyped at 21 autosomal loci and their sex determined by including the amelogenin locus [19]. The samples we selected for this study correspond to 181 unique individuals. Samples collected by the teams of BH and MP were also genotyped at four highly polymorphic loci and their gender determined [2,5].

### Detection of SIVcpz antibodies from fecal samples

All chimpanzee fecal samples were tested for the presence of HIV-1 cross-reactive antibodies using the INNO-LIA I/II score confirmation test (Innogenetics Ghent, Belgium), as described in a previous study [33]. This test contains HIV-1 and HIV-2 recombinant proteins and synthetic peptides coated as discrete lines on a nylon strip. These antigens can cross-react with SIV antibodies present in the sample. We applied dialysis methods previously adopted for antibody detection in fecal samples of gorillas and chimpanzees [2,34]. All assays were performed according to the manufacturer’s instructions and samples were scored as INNO-LIA positive when they reacted with **≥**1 HIV antigen. INNO-LIA-positive samples were also tested by Western blot analysis (Calypte Biomedical; Rockville, MD) as reported previously [2]. RNA*later*^*®*^ precipitated immunoglobulins were resolubilized by diluting fecal/ RNA*later*^*®*^ mixtures (1.5 ml) with PBS–Tween 20 (7.5 ml), followed by inactivation of the mixture for 1 h at 60°C, centrifugation (3500°—g for 10 min) to clarify the solution, and then dialyzing it against PBS overnight at 4°C. The reconstituted extracts were then subjected to immunoblot analysis.

### Distribution models

We used geo-referenced locations of SIVcpz positive sites to model the distribution of the virus using multiple methods. Due to the large number of samples collected at each site, we were also able to determine prevalence of SIVcpz among chimpanzees tested by calculating the number of positives from each location and dividing by the total number of samples tested within the same area. Only sites where 15 or more samples were collected were used in subsequent prevalence analyses. We sought to predict both occurrence and prevalence of SIVcpz in chimpanzees using a suite of environmental variable layers as predictors. Environmental data used included climatic factors involving measures of climate stress such as isothermality and temperature seasonality [35,36], topographic factors such as elevation [37], and measures of the vegetation including the Normalized Difference Vegetation Index (NDVI), a deforestation index [38], and percent tree cover [39]. Pearson Correlation Tests were performed using ENMtools [40] to identify those predictors that explained variation not explained by other environmental layers; highly correlated, redundant environmental variables (r>0.8) not explaining unique variation were then removed.

### Models of SIV occurrence

We used the Maxent distribution algorithm [41], to predict the geographic distribution limits of SIVcpz across the study area. The spatial output of the Maxent distribution model consists of a continuous range indicating the relative probability of occurrence of SIVcpz. Using Maxent, presence localities of SIVcpz were first randomly divided into training (90%) and testing datasets (10%). Models were created using the training dataset, while the testing dataset was used within Maxent to confirm the accuracy of the model after it was created. Final models represent the mean of 100 bootstrapped replicates run by Maxent using random seeds. The bootstrapping replicate process selects training data by sampling with replacement from the entire set of presence localities [42]. Final models were evaluated using the area under the curve (AUC), which is a value widely used to measure model performance [43,44,45]. AUC values were calculated by comparing model performance to a random model of associations between presence localities and environmental variables [45]. AUC values range from 0.5 to 1.0; with values close to 0.5 corresponding to a model that is no better at predicting an ecological niche than a random model, and a value of 1.0 corresponds to a model with a perfect fit. Values greater than 0.9 are”very good”, 0.7–0.9 are “good”, and less than 0.7 are “uninformative” [46]. A jackknife test was also performed using Maxent to evaluate the contribution of each environmental variable to the final model. In the jackknife test, the importance of each factor is tracked while the model is being created by comparing the gain from models with one environmental predicting variable removed at a time to the gain of the complete model with all environmental variables included. The factors that reduce the overall gain of the model when excluded are important in the overall model [41]. An optimized threshold of suitability values greater than 0.2 was applied to final models from Maxent in order to obtain a presence/absence map of infections, which was then used to define the spatial limits for all prevalence predictions.

### Models of SIV prevalence

Random forests [47] were constructed using the package *randomForest* [48] in the R statistical framework (R Statistical Package 2011) to determine the environmental variables that best explained variation in prevalence of SIVcpz in chimpanzees across the study region. A random forest represents a non-parametric binary recursive partitioning approach that attempts to explain variation in a response variable by using each predictor to split the response variable in a manner that minimizes deviance within each ‘bin.’ Predictors are tested in succession, and those that split the response variation best are considered more important in each model. In each test of a random forest run (a tree), records and predictor variables are randomly selected for testing and training purposes; thus the results of random forest represent iterations across multiple independent, unique runs [47]. For random forest analyses, we tested 5,000 trees and measures of variable importance of each predictor tested to arrive at final models. Models explaining the most variation in SIVcpz prevalence were then used to project estimates of prevalence across the study region and were compared to observed values at each study site. These predictions were made by selecting 20,000 random points within the study area and then by extracting predictor values for each variable at those sites. The best random forest model (the one that explained the most OOB, or ‘out of bag,’ variation in the response [48]) was used to predict SIVcpz prevalence at each one of these random points. We then used ordinary kriging [49] to create a continuous prediction of SIVcpz prevalence across the study region.

## Results

### SIVcpz prevalence across Cameroon

Results show that none of the 181 newly collected *P*. *t*. *ellioti* fecal samples tested positive for SIV by INNO-LIA I/II confirmation test. For *P*. *t*. *troglodytes*, 90 out of 1143 samples collected were SIV positive by INNO-LIA I/II confirmation test and western blot analysis (Table 1). Across regions, prevalence varied substantially, ranging from 1.6% (95% CI) in southwest Cameroon to 11% in the center-south and up to 31.6% in the southeast. In 13 locations south of the Sanaga no SIVcpz infections were found, although a total of 390 samples were collected (Table 1 and Fig 2).

**Table 1:** SIVcpz and SFVcpz prevalence by sampling location.

Collection site^a^	Subspecies Origin^c^	Chimpanzee Samples tested for SIVcpz	SIVcpz Antibody-Positive Samples	SIVcpz-Infected Chimpanzees^d^	SIVcpz Prevalence (95% CI)	Chimpanzee Samples tested for SFVcpz^e^	SFVcpz Prevalence (95% CI)^e^
Bankim (BK)	*P*. *t*. *e*	2	0	0	0	0	-
Deuk (DK)	*P*. *t*. *e*	3	0	0	0	0	-
Ebo Forest (EB)	*P*. *t*. *e*	19	0	0	0	0	-
Kombe (KM)	*P*. *t*. *e*	7	0	0	0	0	-
Liabelem Highlands (ED)	*P*. *t*. *e*	3	0	0	0	0	-
Linte (LN)	*P*. *t*. *e*	5	0	0	0	0	-
Makombe (MK)	*P*. *t*. *e*	3	0	0	0	0	-
Mamfe (MF)	*P*. *t*. *e*	39	0	0	0	13	98
Mbam et Djerem (MD)	*P*. *t*. *e*	9	0	0	0	0	-
Metep (MP)	*P*. *t*. *e*	15	0	0	0	5	100
Mone (YW)	*P*. *t*. *e*	4	0	0	0	0	-
Mount Golep (MG)	*P*. *t*. *e*	15	0	0	0	0	-
Ngambe-Tikar (NT)	*P*. *t*. *e*	5	0	0	0	0	-
Takamanda (TK)^b^	*P*. *t*. *e*	1	0	0	0	0	-
Vome (VM)	*P*. *t*. *e*	6	0	0	0	0	-
Wassa Emtse (WE)^b^	*P*. *t*. *e*	25	0	0	0	26	81
Wouchaba (WC)	*P*. *t*. *e*	6	0	0	0	0	-
Yagba (YB)	*P*. *t*. *e*	14	0	0	0	0	-
Belgique (BQ) ^b^	*P*. *t*. *t*	142	0	0	0	82	44
Biwali (BI) ^b^	*P*. *t*. *t*	1	0	0	0	0	-
Bouamir (BM) ^b^	*P*. *t*. *t*	38	2	1	4.9	0	-
Boumba Bek (BB) ^b^	*P*. *t*. *t*	34	0	0	0	31	66
Campo Ma'an (CP) ^b^	*P*. *t*. *t*	232	9	2	1.6	10	100
Deng Deng (DD)	*P*. *t*. *t*	9	0	0	0	0	-
Diang (DG) ^b^	*P*. *t*. *t*	29	0	0	0	29	100
Diassa (DI) ^b^	*P*. *t*. *t*	55	0	0	0	0	-
Djoum (DJ) ^b^	*P*. *t*. *t*	17	4	1	11.0	0	-
Douomo Pierre (DP) ^b^	*P*. *t*. *t*	160	17	4	4.7	114	60
Ekom (EK) ^b^	*P*. *t*. *t*	46	6	4	16.3	19	66
Gribi (GB) ^b^	*P*. *t*. *t*	4	0	0	0	0	-
Kagnol (KG) ^b^	*P*. *t*. *t*	15	0	0	0	0	-
Kika (KK) ^b^	*P*. *t*. *t*	1	0	0	0	0	-
Lobeke (LB) ^b^	*P*. *t*. *t*	38	5	5	24.7	16	53
Lomié (LM) ^b^	*P*. *t*. *t*	62	0	0	0		-
Mambele (MB) ^b^	*P*. *t*. *t*	101	31	17	31.6	25	54
Mbinang (MB) ^b^	*P*. *t*. *t*	24	0	0	0	0	-
Mboi (BY) ^b^*	*P*. *t*. *t*	1	1	1	-	0	-
Minta (MT) ^b^	*P*. *t*. *t*	81	10	2	5.4	81	79
Nki (NK) ^b^	*P*. *t*. *t*	8	0	0	0	0	-
Somalomo (SL) ^b^	*P*. *t*. *t*	44	5	1	4.3	0	-

^a^Location of samples collections are shown in Fig 2.

^b^Samples collected by the team of BH and MP (Keele et al. 2006; Van heuverswyn et al 2007).

^c^*P*. *t*. *e*: *Pan troglodytes ellioti*; *P*. *t*. *t*: *Pan troglodytes troglodytes*.

^d^Based on microsatellite analysis results from Keele et al. 2006; Van heuverswyn et al 2007; Mitchell et al. 2015.

^e^ Data from Liu et al 2008.

* Prevalence was not calculated because the number of samples collected was too small.

Our results strongly suggest that SIVcpz infection is absent or very rare in *P*. *t*. *ellioti*. We took a number of approaches to explore our confidence in this assertion and estimated our power to detect low prevalence of infection given our sampling of 181 individuals. To determine the lowest threshold of SIVcpz prevalence that our sample set (n = 181) would be able to detect at least one SIVcpz infection in *P*. *t*. *ellioti*, should have been observed if the prevalence rate was 2% or higher (p <0.026) (Table 2). Using a Bayesian probability metric, we calculated that, with the current sample size of 181, SIVcpz in *P*. *t*. *ellioti* would have had to occur at a prevalence of less than 1.64% for our test to miss a positive sample.

**Table 2:** Binomial probability of SIVcpz occurrence.

	Assumed incidence of SIVcpz		
	Minimum: 0.86%	Average: 1.5%	Maximum: 2.0%
*β*	0.209	0.065	0.026
*Power*	0.791	0.935	0.974

**Fig 3:**
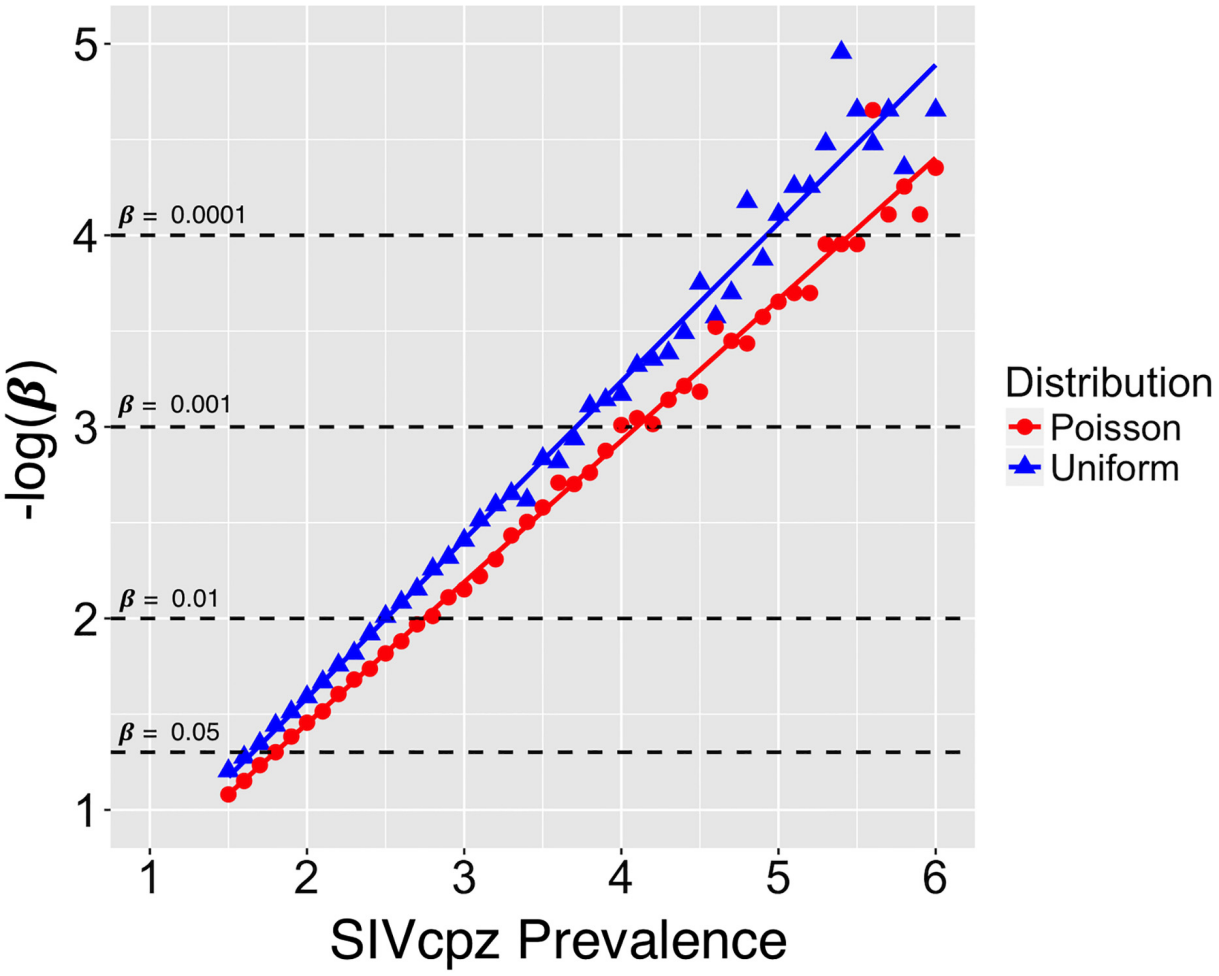
Simulation results estimating ß, the likelihood of erroneously concluding the virus is absent, for a given average prevalence of SIVcpz. Since a single SIV+ case would refute the hypothesis of zero prevalence, ß is equal to the proportion of times zero SIV+ cases are returned when in fact the virus is present. For the uniform distribution (plotted in blue), each data point represents -log_10_ of the proportion of 90,000 simulations returning zero SIVcpz positive cases, assuming each sample site has the same underlying prevalence. For simulations of the Poisson distribution (plotted in red), prevalence is equal to the λ parameter. For each run, sample site prevalence were randomly assigned from sampling the Poisson distribution with a particular λ. A total of 5,000 independent Poisson samples were taken, and the proportion of times zero SIVcpz-positive cases was estimated from 18 binomial sampling runs. Thus, each data point represents the -log_10_ of the proportion of 90,000 simulations returning zero SIVcpz positive cases.

Both estimates suggest an ability to detect the SIVcpz even with a prevalence markedly lower than that found in either *P*. *t*. *troglodytes* or *P*. *t*. *schweinfurthii*. However, both estimates assume a uniform distribution of prevalence among sample sites, and as seen in Table 1, for *P*. *t*. *troglodytes* there is abundant among-site variation in prevalence. We took two approaches to account for potential among-site variation in the prevalence in estimating our ability to detect a single SIVcpz positive individual. In the first, we simulated 10,000 data sets with binomial sampling from the 18 sample sites in *P*. *t*. *ellioti* but assuming prevalence rates present in *P*. *t*. *troglodytes*. Our analysis shows that if SIVcpz was present in *P*. *t*. *ellioti*, with the same among site variance in prevalence as observed in *P*. *t*. *troglodytes*, we would expect to observe no SIVcpz-positive samples 0.4% of the time or less. In our second approach, we assumed that variation in prevalence among sample sites was Poisson distributed. We simulated 5000 data sets with prevalence rates for each of the 18 sample sites for *P*.*t*.*e* sampled from a Poisson distribution with λ (the mean prevalence) ranging from 1.5–6%. For each of the 5000 independent samplings of the distribution we simulated 18 binomial sampling results (a total of 90,000 simulations for each level of λ). For each level of λ we calculated the proportion of runs returning a prevalence of zero across all sites (and thus all 181 individuals, Fig 3). For comparison we also simulated results assuming a uniform distribution of prevalence across the 18 sample sites. While some power is lost as a consequence of among-site variation in prevalence, the effect is negligible. In summary, these various approaches all suggest that we have sufficient power to detect SIVcpz prevalence on the order of 2% or greater in *P*. *t*. *ellioti*. Taken together, these findings suggest that SIVcpz is very rare, if not absent in *P*. *t*. *ellioti*.

### SIVcpz predicted occurrence, prevalence, and environmental correlates

**Fig 4:**
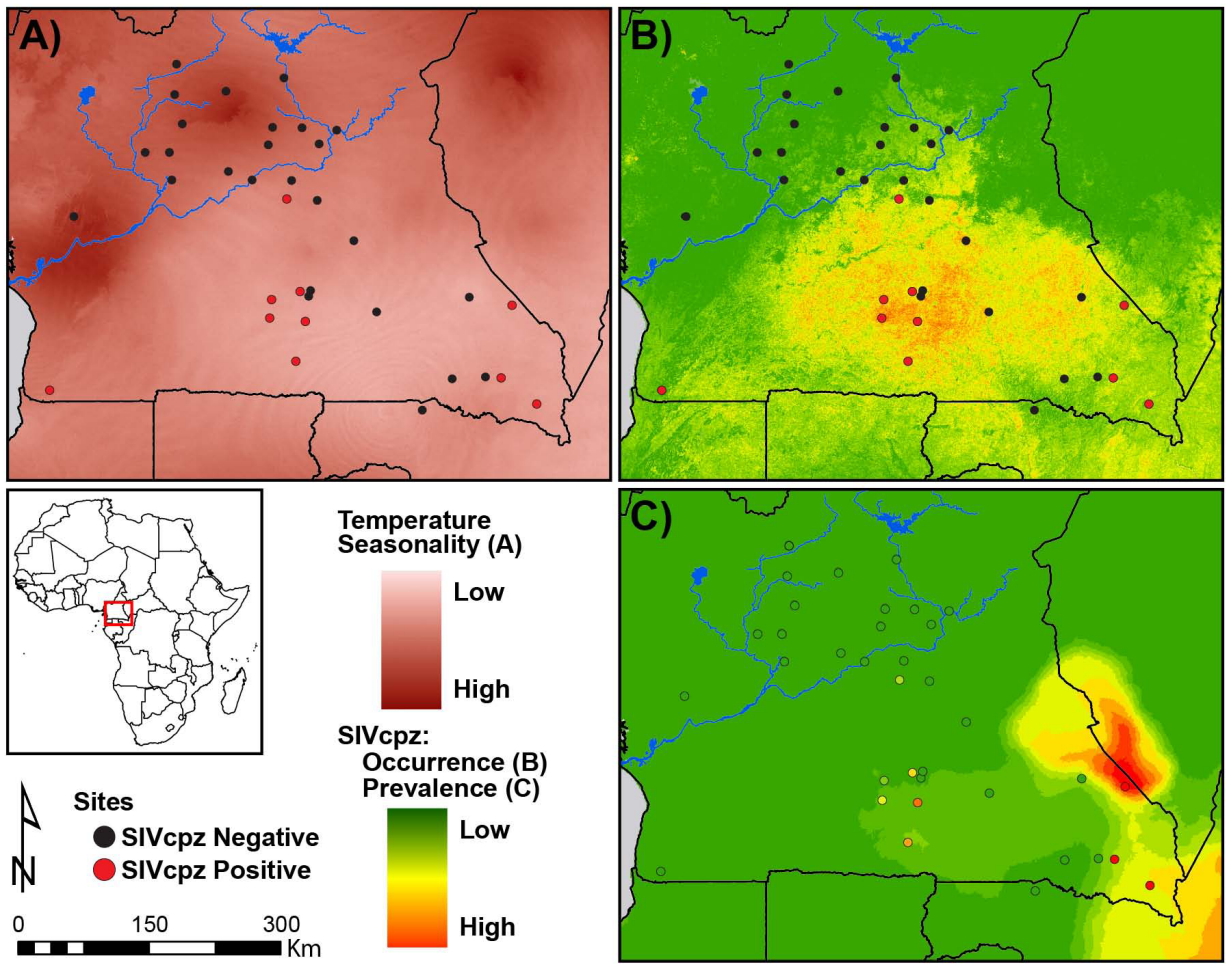
SIVcpz predictions of occurrence and prevalence across Cameroon. (A) Temperature Seasonality, (B) SIVcpz occurrence calculated using Maxent [41], (C) SIVcpz prevalence calculated using *randomForest* [48]. SIVcpz positive sites are shown in red and SIVcpz negative sites are shown in black in A and B. Circles in C correspond to prevalence according to the color ramp to the left.

The initial distribution models for SIVcpz created by Maxent suggest that SIVcpz infection only occurs throughout the forested areas south of the Sanaga River with the highest probability of SIVcpz occurrence in south central Cameroon (Fig 4B). Indeed, some of the locations where we found SIVcpz positive chimpanzees match the highest probability of SIVcpz occurrence as determined by Maxent. This predictive distribution is useful in quantifying the potential ecological niche of SIVcpz-infected chimpanzees, but may not completely capture the realized niche of this species. The most important and significant environmental variable of SIVcpz occurrence was temperature seasonality (Fig 4A), which had the highest predictive contribution (62.4%, S2 Table). Regions within the study area with low temperature seasonality correspond with areas with high estimated SIVcpz occurrence. This relationship can be observed when comparing areas of low SIVcpz occurrence in northwest Cameroon, where temperature seasonality is high, to areas in south and southeast Cameroon, where temperature seasonality is much lower (Fig 4B).

We then obtained a presence/absence map of SIVcpz infection by applying an optimized threshold on the continuous Maxent probability distribution, which we used to define the spatial limits for all prevalence predictions. Prevalence of SIVcpz varied between regions within Cameroon, and this variation was best explained by measures of temperature and precipitation seasonality, explaining up to 32% of the prevalence observed (using only sites where >15 samples were collected). Lower seasonality in both temperature and precipitation were characteristics of regions with high prevalence of SIVcpz infections, in areas such as the central-east through the southeast regions of Cameroon (Fig 4C and S1 Fig). The southeast of Cameroon has been sampled relatively intensively at some locations, with a SIVcpz prevalence of 24.7% (n = 38 fecal samples) for the Lobeke National Park (LB) and 31.6% (n = 101 fecal samples) for the region surrounding the town of Mambele (MB). West of these locations the sites of Ekom (EK) and Djoum (DJ) are inhabited by chimpanzee populations with observed SIVcpz prevalence of 16.3% (n = 46) and 11% (n = 17), respectively. These locations were included in the area where the prevalence predictions determined by RF were moderate to high (Fig 4C). There are not enough data for chimpanzee populations living north of MB and LB, near the border between Cameroon and Central African Republic; further sampling in these regions is required to confirm the high prevalence of SIVcpz predicted by our models.

### SFVcpz predicted occurrence, prevalence, and environmental correlates

**Fig 5:**
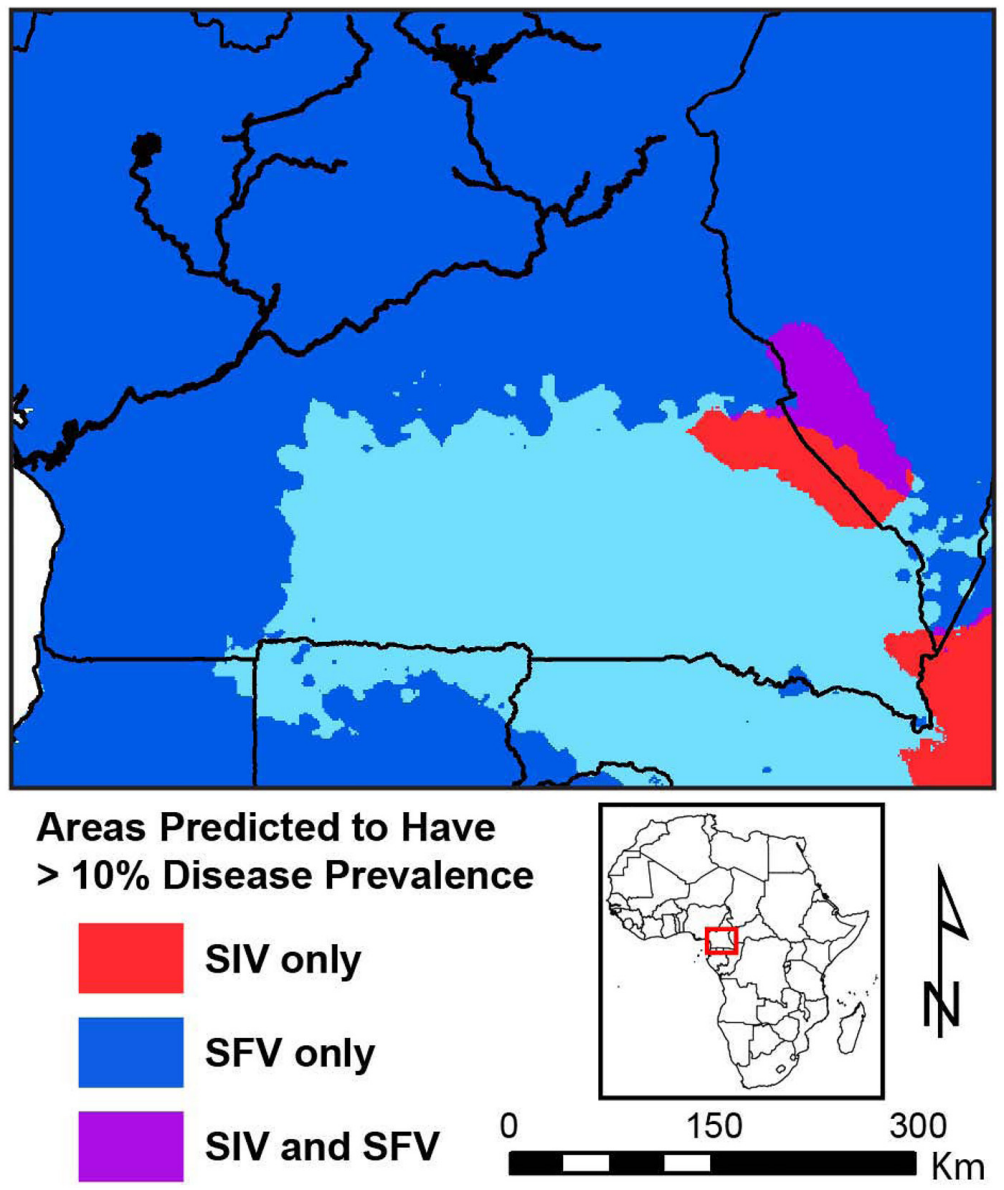
Predicted prevalence of SIVcpz and SFVcpz across Cameroon. Areas with predicted prevalence of >10% include red where only SIVcpz is present; medium blue where only SFVcpz is present; and purple where SIVcpz and SFVcpz overlap. Light blue denotes areas where SIVcpz and SFVcpz may overlap with one another but at a prevalence of <10%.

We ran the models using published data of SFV occurrence and prevalence across this study region [29]. The highest SFV infection rate (~90%) for *P*. *t*. *ellioti* was found at two sites (MF and MP) and for *P*. *t*. *troglodytes* at DG and CP. The lowest infection rate (~60%) was detected in the central south and southeast at the sites of BQ, MB and LB. Maxent and Random forest models performed on SFVcpz occurrence and prevalence indicate that the region with the highest SFVcpz prevalence encompasses the north, center, and southwest of Cameroon and a small area in the southeast neighboring Central Africa Republic (S2 and S3 Figs and Fig 5). Again, seasonality in both temperature and precipitation were important in describing prevalence of infection in chimpanzees, but in this case the trend was opposite as that observed for SIVcpz; higher seasonality of both precipitation and temperature led to higher predicted prevalence of SFVcpz in chimpanzee populations. Measures of seasonality, along with precipitation in the wettest quarter, alone explained 23% of the variation observed across all chimpanzee populations.

### SIV/SFV regression analysis

**Fig 6:**
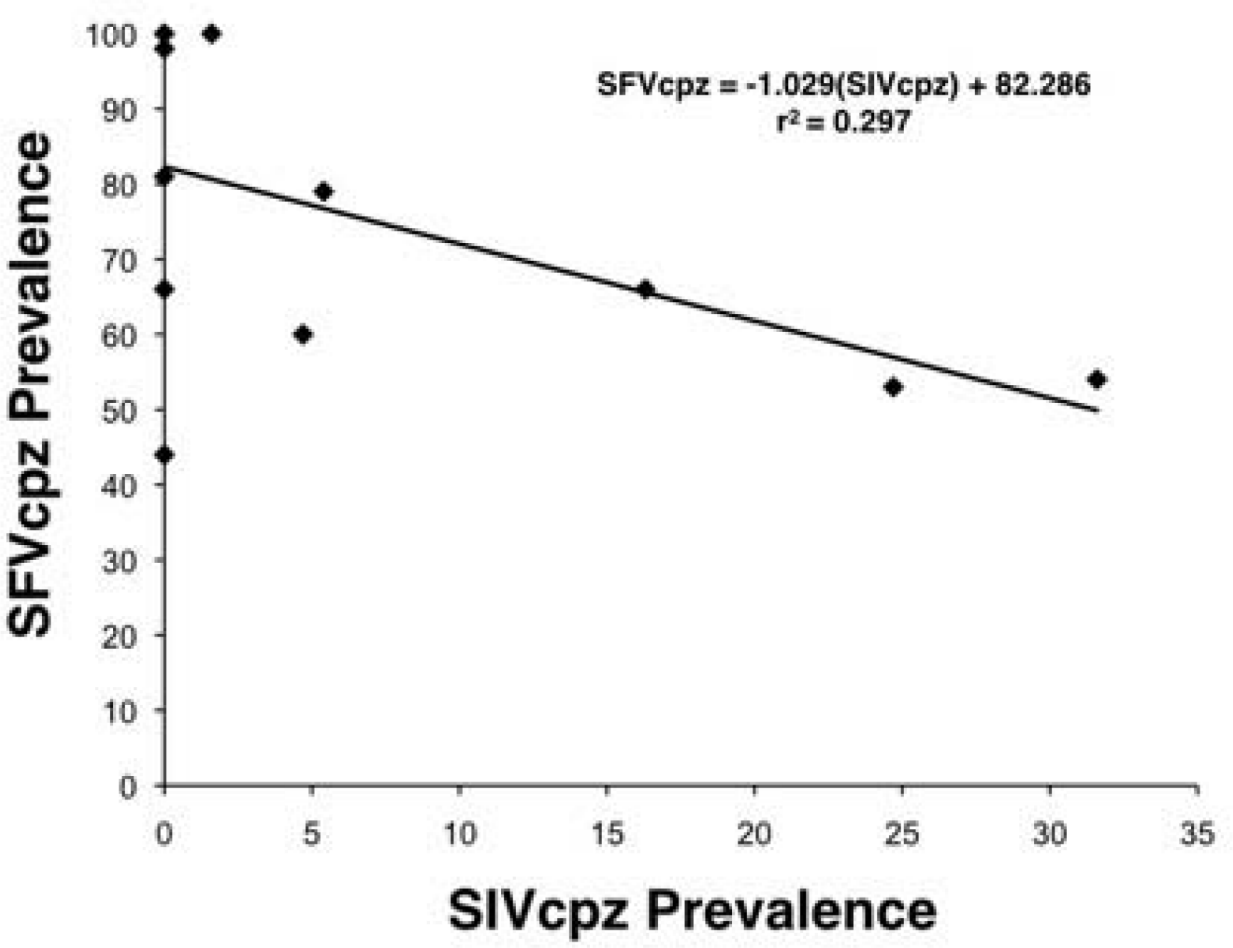
Regression analysis of SFVcpz prevalence on SIVcpz prevalence in *Pan troglodytes ellioti* (3 populations) and *Pan troglodytes troglodytes* (9 populations) from Cameroon. p value = 0.0335 (one-tailed test).

Results from our ecological modeling suggest a negative association between the prevalence of SIVcpz and SFVcpz. For the 12 locations in which fecal samples from both *P*. *t*. *ellioti* and *P*. *t*. *troglodytes* had been analyzed for both viruses, we found a negative relationship between SIVcpz and SFVcpz prevalence (SFV = -1.025(SIV) + 82.2855, r^2^ = 0.297, p = 0.0335, one-tailed test) (Fig 6).

## Discussion

Previous studies reported that *P*. *t*. *ellioti* populations appear to be uninfected by SIVcpz [2,5,32]. What could be the reasons for a lack of SIVcpz infection in *P*. *t*. *ellioti* despite ongoing gene flow between them and SIV infected *P*. *t*. *troglodytes*? One possibility is that *P*. *t*. *ellioti* is actually infected by SIVcpz but at a very low prevalence, or that prevalence varies greatly among locations with those sites with appreciable infection rates having not yet been sampled. In order to rule out this possibility, we extended the sampling area to include more *P*. *t*. *ellioti* chimpanzees, adding 181 samples from 18 different locations north of the Sanaga River. All of these newly collected samples were found to be SIVcpz negative by INNO-LIA HIV confirmation test and Western blot. In addition, power and Bayesian probability analyses suggest that SIVcpz does not occur in *P*.*t*. *ellioti*, or at the very least, is extremely rare compared to adjacent *P*. *t*. *troglodytes* populations. There are several potential explanations for this absence of SIVcpz infections in *P*. *t*. *ellioti*, that center on three main hypotheses: (*i) P*. *t*. *ellioti* has never encountered SIVcpz; (*ii*) members of the subspecies have sporadically encountered SIVcpz but the virus has been unable to sustain an endemic infection; or, (*iii*) *P*. *t*. *ellioti* was endemically infected in the past with subsequent extirpation of the virus in this particular host.

The first hypothesis—that *P*. *t*. *ellioti* has not encountered SIVcpz—seems unlikely for various reasons. Evidence of gene flow between *P*. *t*. *ellioti* and *P*. *t*. *troglodytes* [19] is suggestive of *P*. *t*. *ellioti* exposure to SIVcpz. However, because current genetic data does not provide insight into the amount of gene flow between *P*.*t*.*ellioti* and *P*.*t*.*troglodytes* with respect to the timing of SIV emergence and spread, it is difficult to conclude definitively when SIV may have crossed the Sanaga River, a natural boundary between these two subspecies. SIVcpz prevalence in *P*. *t*. *troglodytes* is highly variable, reaching 30% in some regions but is rare or absent in other areas [2,5,10]. *P*. *t*. *troglodytes* populations closest to the Sanaga River do not appear to be currently endemically infected. However, models suggest that SIVcpz prevalence can fluctuate dramatically with local extinction of the virus [50]. Despite the possibility that the virus is currently absent at or near the Sanaga River and the fact that SIVcpz transmission is rare (approximately of 0.001 per coital act [50]), we cannot discount the fact that the Sanaga river is and it has most probably been a permeable barrier at some point in the past, allowing for genetic flow and possibly disease transmission. We know that SIVcpz was capable of crossing species boundaries on more than one occasion in southern Cameroon [9,51], and that SIVcpz can infect the *P*. *t*. *ellioti* subspecies, as it has been reported in a case of horizontal transmission between a captive male *P*. *t*. *troglodytes* and a captive male *P*. *t*. *ellioti*, [52]. In addition, SIVs and SIV-like ancestors have been infecting other primates likely for thousands to millions of years [53,54,55]: similarly to *P*. *t*. *troglodytes*, *P*. *t*. *ellioti* might have being in contact with a virus while hunting other primates for food [56]. Thus, even if SIVcpz emerged subsequent to the divergence of *P*. *t*. *ellioti* and *P*. *t*. *trogl*odytes, it seems likely that *P*. *t*. *ellioti* individuals would have encountered this virus or an SIVcpz-like virus at some point in their history.

The second and third hypotheses—that *P*. *t*. *ellioti* has encountered SIV, but the virus could not persist and/or went extinct–seem more likely. Both the inability of a virus to establish itself in a new population as well as the extinction of a virus from a population will occur when the basic reproduction number, *R*_*0*_, is less than one. Such reductions in viral fitness could occur as a consequence of increased host immunity, increased virulence, reduced transmission of the virus, or some combination of these factors. Several lines of evidence suggest that one or more of these factors may contribute to the absence of SIVcpz infection in *P*. *t*. *verus* and *P*. *t*. *ellioti*. The absence of SIVcpz in natural populations of *P*. *t*. *verus* [4,6] has been proposed to be due to strong selection imposed by previous SIVcpz-like infections that has resulted in immunity to infection by SIVcpz at present [57,58,59,60]. Recent genetic studies suggest that *P*. *t*. *ellioti* shares a more recent common ancestor with *P*. *t*. *verus* than with *P*. *t*. *troglodytes* [15]. This raises the possibility that—like *P*. *t*. *verus—P*. *t*. *ellioti* also may be immune to SIV infection either as a consequence of a selective sweep driven by an AIDS-like disease prior to its divergence from *P*. *t*. *verus* or that more recent exposure has resulted in the independent evolution of immunity in *P*. *t*. *ellioti*. In both cases, the possibility that a major *P*. *t*. *ellioti* population decline had happened in the past has been excluded in a previous study, which rather reported a stable demographic history of this subspecies [19].

The present strain of SIVcpz appears to be a source of natural selection in *P*. *t*. *troglodytes* and *P*. *t*. *schweinfurthii* because escape from immune control, development of AIDS-like symptoms and deaths have all been documented in natural populations [61,62,63]. The strength of selection is not well understood, but high rates of SIVcpz infection have been associated with rapid population declines in *P*. *t*. *schweinfurthii* [50], which suggests that SIVcpz is a potent agent of selection in affected subspecies. However, these infections appear less pathogenic than HIV-1 infections in human populations [62,64] and certainly not on a level of hypervirulence that could lead to severe reductions in R0. Given the evidence that *P*. *t*. *ellioti* may harbor some intrinsic resistance to SIV, high virulence seems unlikely in this subspecies. Furthermore, a captive *P*. *t*. *ellioti* male that acquired SIVcpz from a *P*. *t*. *troglodytes* cagemate has lived over 20 years without noticeable health problems [52]. Finally, neutral evolutionary forces alone cannot explain the genetic differentiation of *P*. *t*. *ellioti* from *P*. *t*. *troglodytes*, and instead, their differentiation appears to be linked with a pattern of local adaptation [19]. While it is not clear what factors contribute to shaping their divergence from one another, the pattern of a selective sweep at HIV-related loci suggests that adaptation from a past infection might be one reason that *P*. *t*. *ellioti* is not currently infected with SIVcpz. Additional studies looking for signature of selection at genes associated with HIV resistance in *P*. *t*. *ellioti* are needed to test these hypotheses.

Finally, the role of ecological variation in determining occurrence and prevalence of SIVcpz infection may have been previously under-appreciated. Recent research suggests that genetic variation in chimpanzees is linked to differences in ecology [22]. Two genetically distinctive populations of *P*. *t*. *ellioti* occupy two divergent ecological niches in western versus central Cameroon [21]. The western population occupies the mountainous moist habitats of the Gulf of Guinea forest block. The second population occurs in central Cameroon and its distribution coincides with an ecotone comprised of a forest-woodland-savanna mosaic, east of the Mbam River [19,21]. In chimpanzees, differences in environmental conditions such as elevation, climate and vegetation have likely contributed in shaping the genetic diversity found in *P*. *t*. *ellioti* [22]. Thus, we propose that variation in these (and other) environmental conditions, in addition to the Sanaga, might have affected patterns of transmission proximately contributing to the absence and/or extinction of the virus in *P*. *t*. *ellioti*.

Temperature seasonality was a significant environmental predictor of both SIVcpz occurrence and prevalence. Precipitation of the wettest quarter and temperature annual range were also important predictors of SIVcpz prevalence, but not occurrence. Together these environmental variables suggest that higher seasonality in the northwestern portion of Cameroon (north of the Sanaga river) partially explains the differences of SIVcpz occurrence and prevalence. Higher seasonality affects food abundance and distribution, which together with predation pressure and the presence of cycling females in a community, are known factors responsible for shaping differences in social and ecological behaviors [26,65,66,67,68,69]. In particular, population density may influence the number of sexual contacts and partner turnover, due to the sexually promiscuous society of chimpanzees. Females opportunistically mate with multiple males during estrus [65], and SIVcpz spreads primarily through sexual routes [70]. Therefore, migration of infected females constitutes a major route of virus transmission between communities and seems to vary according to the occupied ecological niche. Long term behavioral studies have shown that female transfer is much higher in rainforest than in savanna-forest mosaic ecosystems [65,71,72], although lower female transfer does not always translate into lower rate of SIVcpz infection, as observed for the Gombe communities, where SIVcpz*Pts* prevalence vary from 12 to 46% [73]. Moreover, the number of contacts between different community members is also an important factor influencing the rate of SIVcpz infection, regardless of home range size and population density [10]. Studies on the distribution and behavioral ecology of wild *P*. *t*. *ellioti* are underrepresented [74] compared to the other three *Pan troglodytes* subspecies, but would help to broaden understanding about groups dynamics and consequent disease spread.

To better understand how environmental variation may affect viral pathogens in chimpanzees, we used our ecological modeling to examine the prevalence of SFVcpz. Random forest models suggest that, unlike what was observed for SIVcpz, higher seasonality in both precipitation and temperature lead to a higher predicted SFVcpz prevalence. In fact, our regression analysis demonstrated a marginally significant inverse relationship between SIVcpz and SFVcpz prevalence across populations (Fig 6).

In the study of Liu et al. (2008) the incidence of coinfection with SIVcpz and SFVcpz was examined at four sites across Cameroon (MB, LB, EK, DP) and three sites in Gombe, Tanzania, where both viruses were present. The authors reported that the relative frequencies of single and dual infections at individual sites, or sites in combination, revealed no association between SFVcpz and SIVcpz (Fisher exact test; P = 0.2) [29]. While a reduction in co-infections caused by some mechanism of viral competition could lead to a negative association in viral prevalence among infected populations, this negative association could also arise if each virus is differentially and independently affected by a third variable, such as environmental conditions. Our species distribution models suggest that the ecological niches of these two viruses are, to a large extent, mutually exclusive. Similar to what was observed in chimpanzees, studies on colobines in Uganda and Ivory Coast show that infection with SIV does not increase nor decrease the likelihood of infection with SFV [75,76]. The lack of a combined effect is surprising given that both SIV and SFV infect CD4^+^ lymphocytes. Chimpanzees that develop an AIDS-like disease are prone to suffer from opportunistic infections [62,63]; however we do not know whether SIV-induced immunosuppression will increase the likelihood of development of any disease due to SFV or whether SFV could worsen the outcome of a SIV infection. An experimental study conducted on rhesus macaques showed that there were increases in SIV plasma viral load, in loss of CD4+ T cells, and in animal deaths in pre-existing, natural SFV infected animals as compared to SFV negative animals [77]. Regardless, it is clear that SIVcpz is much less common and widespread among wild chimpanzees than is SFVcpz, similarly to what is observed in other primate species [29]. Although the difference in prevalence of SIVcpz and SFVcpz reported here is only marginally significant, these observations from the same host populations also suggest that these co-circulating retroviruses have different transmission dynamics and exposure routes in natural populations. SIV spreads primarily horizontally, through sexual contact, or in rare cases, by aggression and possibly vertically from the mother to the offspring [70]. In contrast, SFV infection is transmitted mainly through exposure to saliva (or feces) by aggressive behaviors between adults, including biting and possibly grooming at sites of open wounds [28]. Moreover, SIVcpz is potentially harmful [62,63], but there is no indication that SFVcpz is pathogenic in chimpanzees or in other primates [78,79]. Promiscuity encourages the spread of both viruses; however, SFV may circulate more rapidly within a community when males fight each other to mate with receptive females throughout their adult lives, and when frequent aggressive harassment of mating couples occurs. In addition, in highly seasonal regions, where food may be scarce at times, territories would need to be heavily protected, therefore generating more intra and inter-group aggression and increasing further retroviral infection probabilities.

## Conclusions

Results from this study suggest the following: the lack of SIVcpz infection in *P*. *t*. *ellioti* cannot be readily explained by a lack of sampling across the range of this subspecies or by extinction of the virus due to high virulence. These observations leave at least two alternative explanations that should be explored in greater detail in future studies. The first alternative is that *P*. *t*. *verus* and *P*. *t*. *ellioti* may be immune to infection due to a selective sweep from past infections with and SIVcpz-like virus. A handful of loci associated with differences in HIV susceptibility in humans have also been targets of past selection in all chimpanzees subspecies, and limited evidence suggests that selective sweeps at certain loci have occurred in *P*. *t*. *verus* and *P*. *t*. *ellioti* but not in *P*. *t*. *troglodytes* and *P*. *t*. *schweinfurthii*. Secondly, our results suggest that ecological variation is intimately tied to SIVcpz occurrence and prevalence, and that even within a single subspecies of chimpanzee, viral prevalence, transmission probabilities, and ecological conditions favoring such transmissions can vary markedly (Fig 4). Evolutionary and behavioral responses to ecological conditions are known to affect sexual interactions and group dynamics. As with HIV infections in humans, transmission of SIV is primarily sexual. Thus, factors affecting mating behavior will directly affect transmission rates. We propose that environmental and ecological conditions in the range of *P*. *t*. *ellioti* may have shaped factors affecting SIVcpz transmission. Specifically, we speculate that these environmental factors, perhaps in conjunction with relatively low levels of gene flow have contributed to the inability of the virus to establish an endemic infection in this subspecies.

In addition, given the health consequences of SIVcpz, and considering that the probability of occurrence of this virus is estimated to be highest south of the Sanaga River, populations of chimpanzees of Cameroon warrant further monitoring and regular testing for SIVcpz in order to establish the impact of the virus on these populations as well as potential new foci of zoonotic transmission. Even more concerning, though, is that the habitat where both viruses are present is likely to expand in Cameroon according to future climate change predictions. The latest Intergovernmental Panel on Climate Change (IPCC) projections suggest an average three degree Celsius increase in average temperature within Cameroon [80]. Seasonality is an important predictor of SIVcpz prevalence (inversely related), and this variable is predicted to decrease with increasing annual temperatures. With this decrease in temperature seasonality, we can expect the occurrence and prevalence of SIVcpz to increase in chimpanzee populations in this region. Given the myriads of additional stressors to these primate populations, including climate change effects on physiology, increased human land-use activity, and changes to resource availability, it is imperative that we understand how pathogens are currently affecting the chimpanzee populations of Central Africa to better understand the impacts of these pathogens in the future.

## Supporting Information

S1 Fig SIVcpz prevalence calculated using random forest models, taking into account only the sites where more than 15 samples were collected. **SIVcpz** prevalence decreases according to a color gradient, from red (highest) to green (lowest).
(TIF)

S2 Fig Predicted occurrence of SFVcpz across Cameroon. In this output map of a SFVcpz Maxent model projection, colder colors denote areas of low occurrence or absence, warmer colors of highest occurrence.
(TIFF)

S3 Fig Predicted prevalence of SFVcpz across Cameroon. Warmer colors denote areas of highest prevalence, whereas green indicates areas of lower prevalence or absence. Circles represent areas where SFVcpz data were collected [29].
(TIFF)

S1 Table GPS coordinates of the sampling sites. (PDF)

S2 Table Estimates of relative contributions of environmental variables to the Maxent SIVcpz model. (PDF)

S3 Table Open access resources to create maps for Figs 1, 2, 4 and 5. (PDF)
