# Supplementary figures and images for: Why Are Nigeria-Cameroon Chimpanzees (Pan troglodytes ellioti) Free of SIVcpz Infection?
Source: PLoS One. 2016 Aug 9;11(8):e0160788. doi: 10.1371/journal.pone.0160788 (PMC4978404; doi:10.1371/journal.pone.0160788)

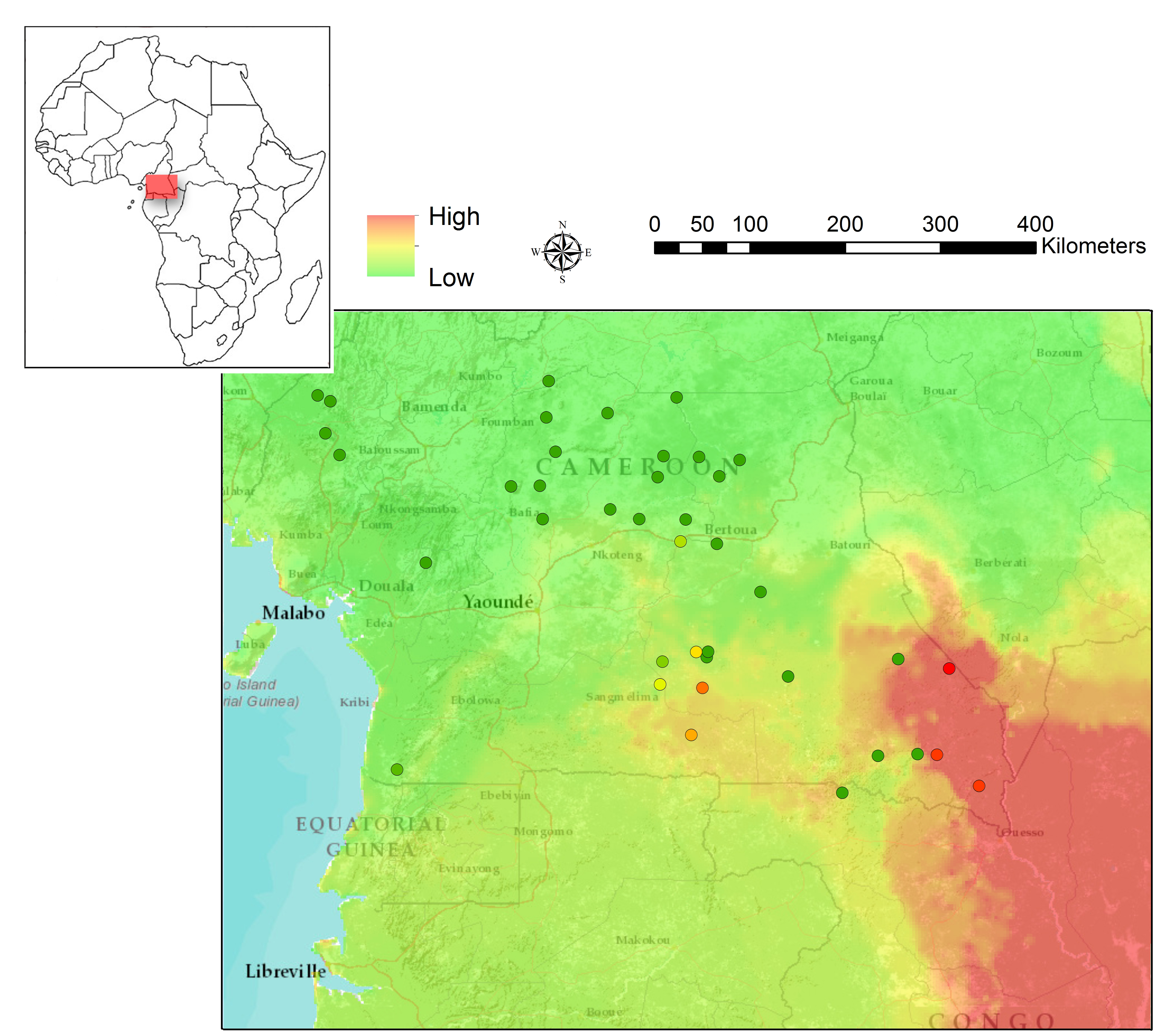

Supplement: S1 Fig — SIVcpz prevalence decreases according to a color gradient, from red (highest) to green (lowest). (TIF) [file pone.0160788.s001.tif]

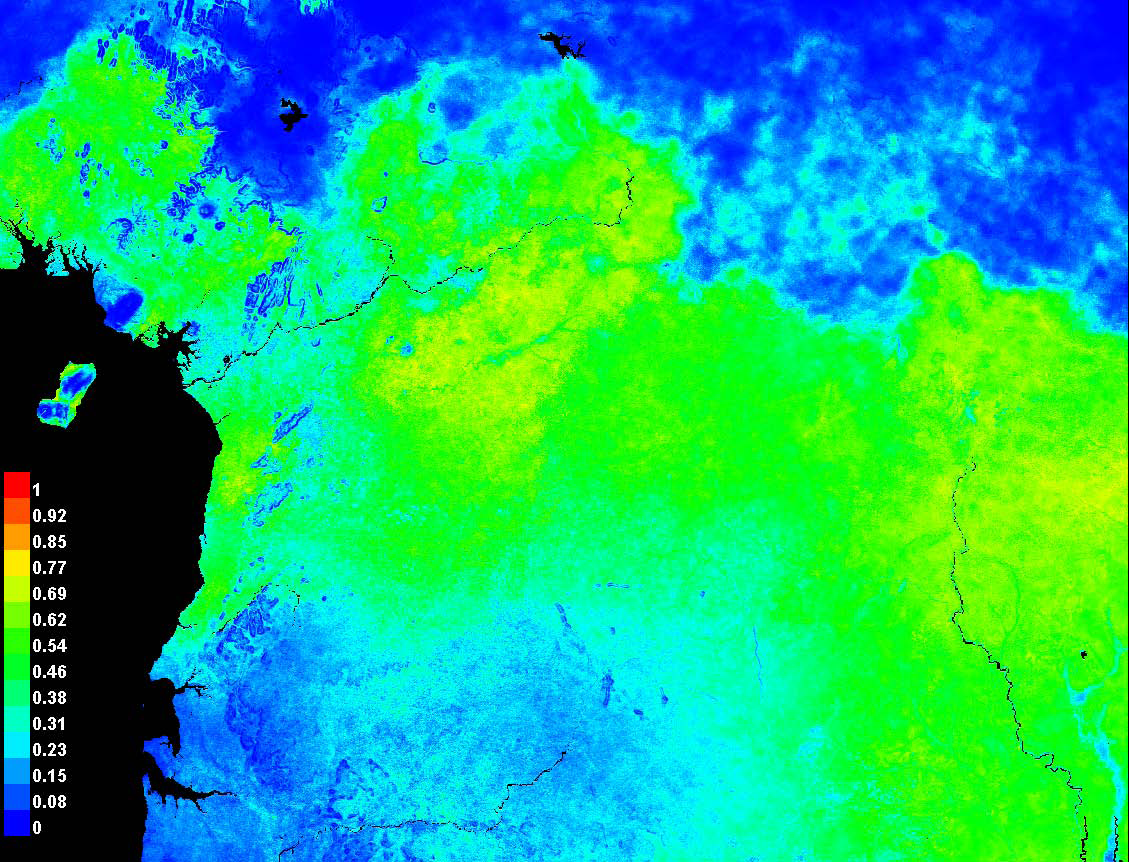

Supplement: S2 Fig — In this output map of a SFVcpz Maxent model projection, colder colors denote areas of low occurrence or absence, warmer colors of highest occurrence. (TIFF) [file pone.0160788.s002.tiff]

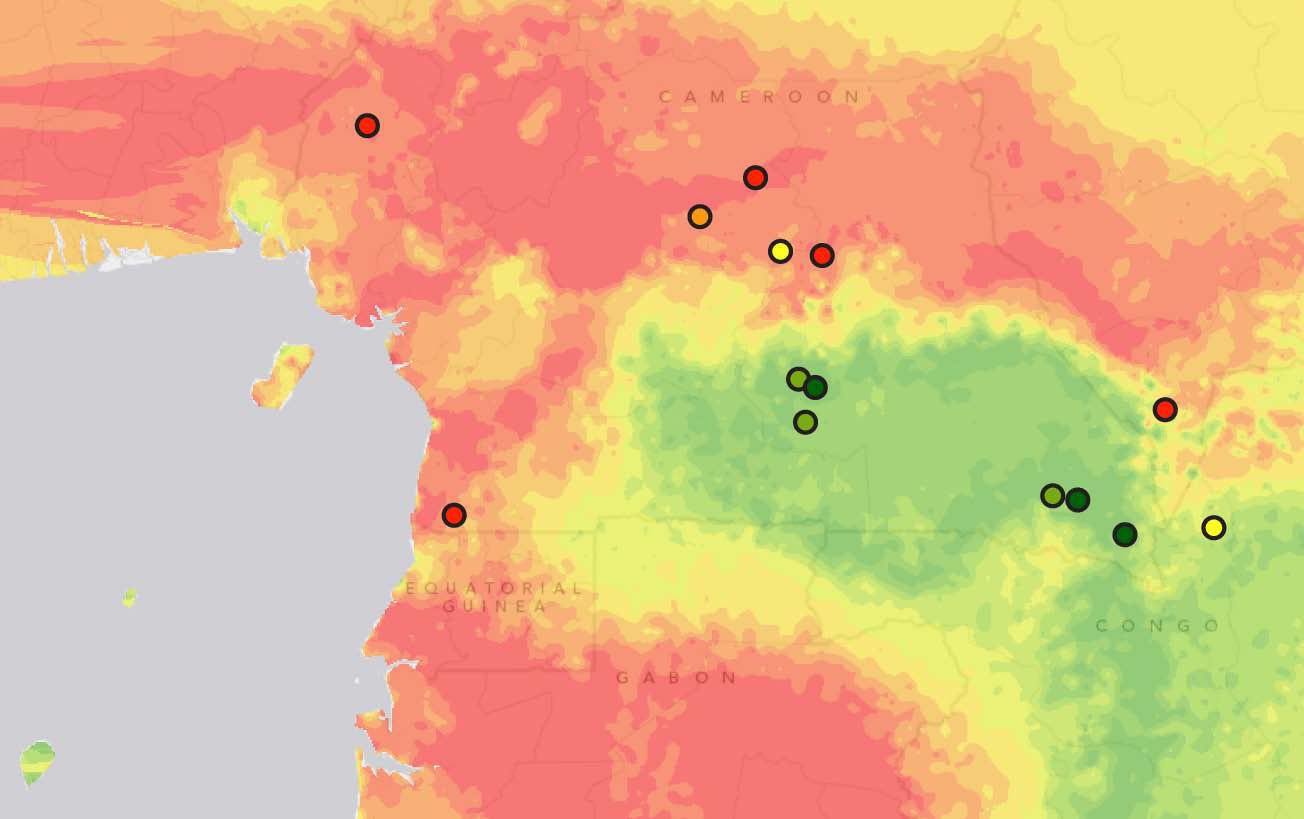

Supplement: S3 Fig — Warmer colors denote areas of highest prevalence, whereas green indicates areas of lower prevalence or absence. Circles represent areas where SFVcpz data were collected [29]. (TIFF) [file pone.0160788.s003.tiff]
