# Supplementary material for: Why Are Nigeria-Cameroon Chimpanzees (Pan troglodytes ellioti) Free of SIVcpz Infection?
Source: PLoS One. 2016 Aug 9;11(8):e0160788. doi: 10.1371/journal.pone.0160788 (PMC4978404; doi:10.1371/journal.pone.0160788)
Supplement: S1 Table — (PDF) [file pone.0160788.s004.pdf]

Sample Location	Sample Abbreviation	Longitude	Latitude
Bankim	BK	11,620949	6,123408
Deuk	DK	11,561027	4,772889
Ebo Forest	EB	10,414740	4,345430
Kombe	KM	13,290114	5,191496
Liabelem Highlands	ED	9,571436	5,399501
Linte	LN	11,687588	5,430429
Makombe	MK	12,744100	5,389500
Mamfe	MF	5,61225	9,43269
Mbam-Djerem National Park	MD	12,874200	5,964100
Metep	MP	12,688986	5,182878
Mone	YW	9,480388	5,927165
Mount Golep	MG	11,251401	5,091681
Ngambe-Tikar	NT	11,596917	5,769220
Takamanda	TK	9,35635	5,98328
Vome Village	VM	12,196700	5,810400
Wassa Emtse	WE	12,223570	4,867510
Wouchaba	WC	13,092472	5,381611
Yagba	YB	11,533904	5,097671
Belgique	BQ	13,169200	3,418400
Biwali	BI	12,506444	4,771389
Bouamir	BM	12,710842	3,150327
Boumba Bek	BB	15,234720	2,468190
Campo Ma'an National Park	CP	10,133447	2,316771
Deng Deng National Park	DD	13,491075	5,351734
Diang	DG	13,2663	4,52883
Diassa	DI	13,1818	3,4699
Djoum	DJ	13,017333	2,655133
Douomo Pierre	DP	13,06459	3,46867
Ekom	EK	13,126094	3,117543
Gribi	GB	15,044092	3,39936
Kagnol	KG	13,694334	4,057325
Kika	KK	14,8465	2,450918
Lobeke	LB	15,836163	2,155906
Lomié	LM	13,963922	3,228114
Mambele	MB	15,42361	2,46066
Mbinang	MG	12,962179	4,767626
Mboi	BY	15,544659	3,307151
Minta	MT	12,912	4,55219
Nki	NK	14,49703	2,08956
Somalomo	SL	12,73344	3,37315
