# Supplementary material for: Why Are Nigeria-Cameroon Chimpanzees (Pan troglodytes ellioti) Free of SIVcpz Infection?
Source: PLoS One. 2016 Aug 9;11(8):e0160788. doi: 10.1371/journal.pone.0160788 (PMC4978404; doi:10.1371/journal.pone.0160788)
Supplement: S2 Table — (PDF) [file pone.0160788.s005.pdf]

Type of Layer	Environmental Layer	Percent Contribution
Topographic	Altitude	0.7
	Ruggedness	**5.2**
Climatic	Temperature Seasonality (Bio4)	**62.4**
	Maximum Temp. of the Warmest Month (Bio5)	**7.4**
	Temperature Annual Range (Bio 7)	1.6
	Precipitation of the Warmest Quarter (Bio18)	0.8
	Annual Mean of Surface Moisture Content (QSCAT)	5.1
	Surface Moisture Content Variation (QSCATSTD)	1.7
Vegetation	Tree Cover	0.2
	Enhanced Vegetation Index (EVI)	3.2
	Maximum Normalized Difference Vegetation Index (NDVI) of Least Green Season (NDVIBR)	3.7
	NDVI Seasonality (NDVIGRBR)	1.1
	Rainforest Deforestation Index (RFDI)	1.6
	MODIS Band 1	1.7
	MODIS Band 7	3.5

†Bold values represent about the top 75% of contributing variables for the model.
