# Supplementary material for: Why Are Nigeria-Cameroon Chimpanzees (Pan troglodytes ellioti) Free of SIVcpz Infection?
Source: PLoS One. 2016 Aug 9;11(8):e0160788. doi: 10.1371/journal.pone.0160788 (PMC4978404; doi:10.1371/journal.pone.0160788)
Supplement: S3 Table — (PDF) [file pone.0160788.s006.pdf]

- **Figure 1**

Chimpanzee ranges - IUCN International Union for conservation of Nature (IUCN) at http://www.iucnredlist.org/details/15933/0. Bonobos ranges are from IUCN at http://www.iucnredlist.org/details/15932/0.
The background layer is “percent tree cover” from the MODIS satellite, from Hansen et al. (2000), Global land cover classification at 1 km spatial resolution using a classification tree approach. *International Journal of Remote Sensing*. Vol.21, Iss.6-7.
Country borders are from GADM- Global Administrative Areas. (2012). "GADM level 0 dataset." Retrieved October 2015 from http://www.gadm.org/.

- **Figure 2**

Chimpanzees ranges- IUCN International Union for conservation of Nature (IUCN) at http://www.iucnredlist.org/details/15933/0.
Background layer is “percent tree cover” from the MODIS satellite, from Hansen et al. (2000)
Rivers are from the HydroSHEDS hydrography layer from Lehner et al. (2008), *Eos, Transactions, American Geophysical Union*,Volume 89, Number 10, 4 March 2008. Eos Trans. AGU, 89: n/a. doi:10.1029/eost2008EO10
Country borders are from GADM - Global Administrative Areas. (2012). "GADM level 0 dataset." Retrieved October 2015 from http://www.gadm.org/.

- **Figure 4**

Country borders are from GADM - Global Administrative Areas. (2012). "GADM level 0 dataset." Retrieved October 2015 from http://www.gadm.org/.

- **Figure 5**

Country borders are from GADM - Global Administrative Areas. (2012). "GADM level 0 dataset." Retrieved October 2015 from http://www.gadm.org/.
